# Life review therapy for holocaust survivors (LRT-HS): study protocol for a randomised controlled trial

**DOI:** 10.1186/s12888-020-02600-5

**Published:** 2020-04-25

**Authors:** Simon Forstmeier, Elisheva van der Hal, Martin Auerbach, Andreas Maercker, Danny Brom

**Affiliations:** 1 Developmental Psychology and Clinical Psychology over the Lifespan, Institute of Psychology, University of Siegen, Adolf-Reichwein-Str. 2a, 57068 Siegen, Germany; 2 Amcha, The National Israeli Center for Psychosocial Support of Survivors of the Holocaust and the Second Generation, 23 Hillel Str., P.O. Box 2930, 91029 Jerusalem, Israel; 3 Psychopathology and Clinical Interventions, Department of Psychology, University of Zurich, Binzmuehlestr. 14/17, 8050 Zurich, Switzerland; 4 Herzog Hospital, Givat Shaul St, 91035 Jerusalem, Israel; 5 Hebrew University Jerusalem, The Paul Baerwald School of Social Work and Social Welfare, Jerusalem, Israel

**Keywords:** Post-traumatic stress disorder, Depression, Prolonged grief, Old age, Holocaust survivors, Life review therapy, Reminiscence, Narrative exposure, Randomised controlled trial

## Abstract

**Background:**

The Holocaust was one of the most traumatic catastrophes in recorded human history. Survivors seeking psychotherapeutic help today, now in their seventies and older, often show symptoms of a posttraumatic stress disorder (PTSD), depression, or prolonged grief disorder. Established psychological treatments for PTSD (e.g. cognitive behaviour therapy, psychodynamic therapies) have been tested and assessed mainly with young and middle-aged adults; only very few studies examined them in old age. There is no therapy outcome study known to us for any treatment mode for Holocaust survivors. Moreover, there is a need for an age group-specific treatment of PTSD and other stress-related mental disorders. A narrative approach including life-review and narrative exposure seems to meet very well the natural need of older people to review their lives and is highly effective. However, most studies on the efficacy of life review therapy (LRT) focus on late-life depression. There is a lack of efficacy studies evaluating the effect of LRT on PTSD symptoms in older individuals that have experienced traumatic events.

**Methods:**

The main goal of this study is to evaluate the effect of LRT for Holocaust survivors (LRT-HS) on symptoms of PTSD and related mental health problems (depression, anxiety, prolonged grief), compared to a supportive control group. A secondary goal is to identify the characteristics of participants that seem to especially benefit from the treatment. The proposed study is a randomised, controlled follow-up trial including Holocaust survivors with one or more trauma-related disorders. The LRT treatment consists of 20–25 sessions. Before and after the treatment phase, participants in both conditions will be assessed. Follow-up will take place 6 months after the treatment. A sample size of 80 is required (drop-out rate included).

**Discussion:**

Efficacious treatments for trauma-related disorders in older people are of high importance, also because the probability of traumatisation and loss increases with age. Because this study is conducted with this specific group of multiply traumatised people, we are convinced that the results can easily transfer to other samples.

**Trial registration:**

ISRCTN, ISRCTN12823306. Registered 31 March 2018 – Retrospectively registered (first participant 22 December 2017).

## Background

### The holocaust and its psychological consequences in old age

The Holocaust was one of the most traumatic catastrophes in recorded human history. Over the span of several years, a multitude of traumatic events were experienced by a large group of people: persecution and flight under threat of death, loss of family members, starvation, fighting, rape, forced displacement, concentration or extermination camps, torture, witnessing torture and death and being forced to survive in hiding etc. The majority of Holocaust survivors alive at present (now in their seventies and eighties or older) were children and teenagers during the years of the Holocaust. In addition to experiencing traumatic stressors, being a child or adolescent before, during and even after the Second World War (WWII) also meant experiencing massive losses and being separated from parents, relatives and home [1].

Survivors grown old, seeking psychotherapeutic treatment today, exhibit a deterioration of the capability to deal with the burden of the past by a depressed, or irritable mood as a result of an underlying posttraumatic stress disorder (PTSD). The mental health of Holocaust survivors is in average still worse today than a comparison group, mainly with regard to PTSD symptoms, depression and anxiety [2]. The majority of Holocaust survivors show a chronic course, i.e. they experience a steady decrease of PTSD symptom severity, whereas about 10% of survivors show a delayed course, i.e. symptoms increase over time or get reactivated after a long symptom-free time [3].

Although Holocaust survivors exhibit incredible adaptive processes [4], aging poses new challenges for this adaptation process. Aging is accompanied by reviewing one’s life in order to evaluate gains and losses and find meaning [5]. When old people engage in retrospection, past memories might evoke those early experiences of trauma, loss and deprivation and induce or intensify symptoms of PTSD, anxiety, depression and grief. In addition, confrontation with new crises (e.g. retirement), losses (persons, resources, physical and mental decline) and traumatic events (e.g. wars) might further conjure up those childhood memories and intensify symptoms [6]. Living in Israel is especially characterised by a constant feeling of danger and looming threat due to the political instability and ongoing threat of war in the Middle East.

### State of treatment research

#### Established PTSD treatments

Most PTSD treatment research has focused on children and young and middle-aged adults. The need for psychotherapy for the elderly, especially for those who suffer from PTSD, has not been subject to systematic study up until very recently [7]. Established psychological treatments for PTSD can be roughly divided into (a) trauma-focused approaches including exposure and cognition-focused approaches (e.g. cognitive behaviour therapies, CBT, eye movement desensitisation and reprocessing and narrative exposure) and (b) approaches with a broader focus, i.e. not only on the trauma, but also on associated problems such as guilt, anger, grief and depression (e.g. psychodynamic therapies, PDT and non-directive counselling). In addition (c), trauma-focused (i.e. cognitive and behavioural) interventions are often integrated into a psychodynamic treatment.

RCTs on PTSD treatment in old age are very scarce. We identified two RCTs that examined this age group. An internet-based writing therapy called Integrative Testimonial Therapy (ITT) with WWII survivors combining cognitive-behavioural and life review elements found moderate decreases in PTSD symptoms [8, 9]. A prolonged exposure therapy with older veterans with PTSD as a result of their military service found treatment gains from pre- to posttest, but those gains were lost in the follow-up [10, 11].

Narrative approaches integrate exposure and cognitive interventions [e.g., [12]], their efficacy has been proven [13], but not in old age so far. Narrative approaches aim at reconstructing the memories of traumatic events and transforming them into a coherent narrative (exposure focus), integrating those memories into the biography and reassessing the meaning of the events (cognition focus).

#### Treatment of holocaust survivors in Israel

The most often offered treatment of Holocaust survivors in Israel is based on a psychodynamic understanding which integrates trauma-focused elements, i.e. imaginary or behavioural exposure, cognitive reappraisal and narrative techniques [14]. “Brief psychodynamic therapy” [15] is very often applied in the treatment of trauma patients. One randomised controlled trial (RCT) *without* older patients showed that it is more effective than the waiting list control group and similarly effective as trauma-focused therapy [16]. However, the treatments that have been shown to be effective in early and middle adulthood have just started to get systematically investigated in old age, and there is no randomised, controlled study with Holocaust survivors.

Amcha, the “National Israeli Center for Psychosocial Support of Survivors of the Holocaust and the Second Generation”, is a pioneer in helping and treating Holocaust survivors, and adapted an integrative psychodynamic approach [17]. Amcha has been founded in 1987 by a group of Holocaust survivors and mental health professionals with the goal (as a non-profit organisation) to support and treat survivors and their families with psychosocial interventions (mainly psychotherapy and social clubs). Amcha has steadily expanded over the 30 years of its existence. Today there are 15 centres in Israel that operate throughout the country. Most of the professionals are trained psychotherapists – psychiatrists, psychologists and social workers. About 8700 Holocaust survivors are currently treated by Amcha. Given the lack of efficacy studies of psychodynamic PTSD treatment in old age, it is needless to state that the efficacy of the treatment offered at Amcha has not been investigated so far.

#### Life review therapy as an age-specific treatment of PTSD

One approach to the treatment of *older* PTSD patients is to apply the established psychological therapies described above with minor adaptations [e.g., [11, 18]]. However, the question arises whether an age-specific treatment of PTSD would be more adequate. Aging is accompanied with reviewing one’s life in order to evaluate gains and losses and to find meaning [5]. Therefore, a narrative approach as mentioned above seems to meet very well the natural need of older people to review their lives [19]. Most studies on the efficacy of life review therapy (LRT) focus on late-life depression. A meta-analysis comparing the effect of life-review, CBT, PDT and other treatments on clinically depressed older adults found large effect sizes for LRT and CBT and medium effect sizes for PDT [20]. However, there is a lack of efficacy studies evaluating the effect of life-review therapy on PTSD symptoms in older individuals that have experienced traumatic events during their lives [19]. This is particularly surprising since there are very good experiences with narrative approaches in the treatment of PTSD with children, adolescents and young and middle-aged adults [13]. Maercker [21] combined the life-review therapy approach [22] with narrative exposure to the traumatic events such as in narrative exposure therapy [12]. He reported on three case studies that found this life-review therapy to be effective in German survivors of the Nazi period [21]. Building on these case studies, Knaevelsrud et al. [8] developed the Integrative Testimonial Therapy (ITT) approach, an internet-based writing therapy that integrates life-review therapy with narrative exposure and cognitive restructuring. Preliminary results of a pre-post follow-up study with a sample of older PTSD patients who were traumatised as children during WWII indicate that this therapy significantly reduces PTSD and anxiety symptoms.

While there is no controlled study on the efficacy of LRT with Holocaust survivors, experimental and narrative research on life stories of Holocaust survivors supports the assumption that it would be effective in this sample. For example, it was shown that the degree of disclosure during the telling of their story was positively correlated with long-term health [23]. Furthermore, in a longitudinal case study, one Holocaust survivor was systematically interviewed twice, in 1982 and 1995 [24]. It was found that while structure and general content of the narratives remain highly consistent over time, the later narrative was more fully developed and integrated new interpretations as well as historical insights. This qualitative finding suggests that Holocaust survivors might be open for re-evaluating their lives when reviewing major events.

### The need for a trial

From the previous literature review follows that not only the traditional, age-unspecific approaches (PDT, CBT), but also the age-specific LRT have not been evaluated with state-of-the-art methodology in Holocaust survivors. Therefore, an RCT that investigates the efficacy of LRT (as an age-specific trauma treatment) is urgently needed [25].

### Study objectives

To our knowledge, there is no controlled study evaluating the efficacy of psychotherapy in Holocaust survivors. Therefore, the general goal of this study is to evaluate the efficacy of an integrative, age-specific treatment of PTSD and related mental health problems (depression, anxiety, prolonged grief) in Holocaust survivors.

From the previous research outlined above follows that LRT, which not only meets the natural needs of older people to review their lives, but is also effective in the treatment of depression (as shown by several RCTs) and PTSD (as shown by case studies and preliminary results from one RCT), seems to be most promising for the treatment of Holocaust survivors.

Part of the basic support structure of Holocaust survivors in Israel are social support services, which are also provided by all Amcha centres throughout the country (called “social club”). The social clubs are meant to let survivors come together in an environment where they meet people with whom they share common experiences now and then. The clubs provide activities like films, lectures, courses in art, music, memory improvement, gymnastics, languages (English, Yiddish), visits to interesting places, birthday celebrations, discussions of relevant issues, etc. They are not intended for psychodynamic or trauma-focused psychotherapy.

Thus, in this study, participants receiving the treatment (LRT) are explicitly allowed to continue visiting the social club. The control group consists of social club member who receive no psychotherapeutic treatment (LRT or other). Control group participants might receive LRT after the follow-up assessment.

The goals of this study are:

1. to evaluate the effect of LRT for Holocaust survivors (LRT-HS), in addition to attending the “social club”, on symptoms of PTSD and related mental health problems (depression, anxiety, prolonged grief), compared to a supportive control group (“social club”);
2. to identify groups of participants that seem to especially benefit from the treatment.

Two research questions will be investigated:

1. Does the LRT-HS as short-term treatment for Holocaust survivors, in addition to attending the “social club”, reduce the symptoms of PTSD and related mental health problems (depression, anxiety, prolonged grief) more than a supportive control group (only attending the “social club”)?

We expect that life-review therapy will reduce the symptoms of PTSD and related mental health problems with a large pre-post effect size and to a greater extent than the supportive control group at posttest and 6-months follow-up with a medium-to-large effect size.

2. Which factors moderate the treatment effect, i.e. which factors influence who benefits most from the intervention?

We expect that LRT-HS will have a greater effect in people (a) with more severe symptoms of PTSD, depression, anxiety and/or grief at pretest, (b) with lower positive well-being and posttraumatic growth at pretest, (c) of female gender, (d) with higher cognitive status at pretest, (e) with higher trauma severity, i.e. number of traumatic events, months/years experienced during the Holocaust, as well as traumatic experiences before and after the Holocaust.

## Methods/design

### Design

The proposed study is a randomised, controlled follow-up trial including Holocaust survivors with one or more stress-response disorders (i.e. PTSD, depression, anxiety and/or prolonged grief).

**Fig. 1:**
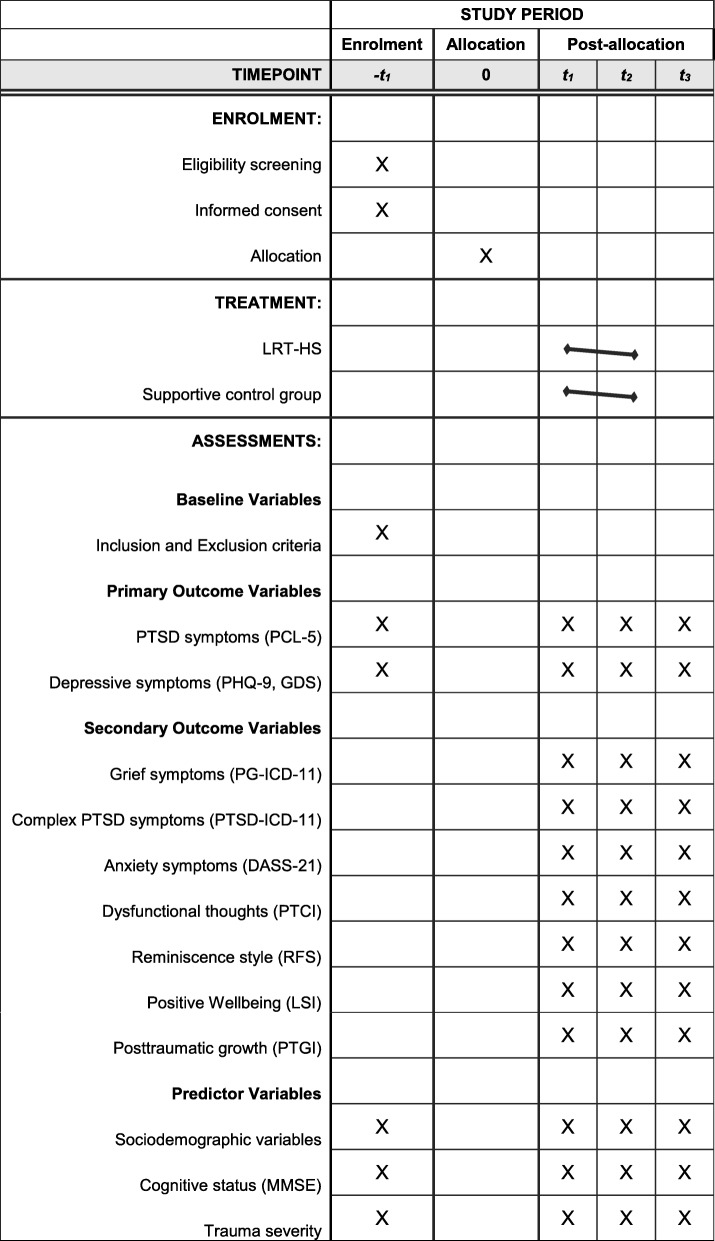
SPIRIT schedule of enrolment, treatment, and assessments. Abbreviations: DASS-21: Depression Anxiety Stress Scale-21; GDS: Geriatric Depression Scale; LSI: Life Satisfaction Index; MMSE: Mini Mental Status Examination; PCL-5: PTSD Checklist for DSM-5; PHQ-9: Patient Health Questionnaire 9; PTGI: Posttraumatic Growth Inventory; PTCI: Posttraumatic Cognitions Inventory; PTSD: Posttraumatic stress disorder; RFS: Reminiscence Functions Scale

Participants will be randomly assigned (see Fig. 1) to either the LRT (in addition to the social club) or a supportive control group (social club only). The treatment includes 20–25 sessions. The participant timeline is presented as SPIRIT schedule in Fig. 1.

Enrolment into one of the two groups will be done consecutively during the recruitment phase. The treatment phase takes approx. Nine months. Before and after the treatment phase, participants in both groups will be assessed. Follow-up will take place at 6 month post-treatment. All assessments will be conducted by blind assessors.

### Sample

The sample consists of Holocaust survivors, i.e. Israeli individuals who were born in 1945 or earlier in Europe and experienced persecution, concentration camp, witnessed torture and death, or who survived in hiding and/or who experienced other traumatic events by the Nazi regime during WWII.

Each participant is diagnosed with either full or subsyndromal PTSD, depressive disorder (major depression, minor depression), anxiety disorder, or prolonged grief disorder.

#### Recruitment

The participants will be recruited from ten centres of Amcha in Israel. Mean age of Holocaust survivors attending the social club at Amcha centres is currently (in the year 2020) 89 years, the youngest were born in 1945. About 30% were born between 1935 and 1945 (now 75–85 years old), 50% between 1925 and 1935 (now 85–95 years old) and 20% were born before 1925 (now over 95 years old).

Study participants will be recruited from current and new social club members. During the time of the research project, there will be active advertisements in the community and at medical practices in order to introduce Amcha, its social clubs and the study treatment. The fact that Amcha is present in 15 cities across Israel, has a very good reputation in the country, and is working with other medical institutions, makes the proposed study highly feasible.

#### Inclusion and exclusion criteria

Inclusion criteria are:

- Being a Holocaust survivor, i.e. born in 1945 or earlier in Europe and having experienced persecution, concentration camp, witnessed torture or death, or been forced to survive in hiding and/or experienced other traumatic events by the Nazi regime during WWII.
- Diagnosis of full or subsyndromal PTSD, depressive disorder (major depression, minor depression), anxiety disorder, or prolonged grief disorder.
- Participants do not receive any individual psychotherapy during the time of the study (except for LRT in the intervention condition).
- Participants must have been informed about the study and must have given written informed consent. This includes that he/she is willing to be randomly assigned to one of the study groups.

Exclusion criteria are: Probable dementia (Mini Mental Status Examination, MMSE < 26); acute psychotic disorder; acute suicidality.

#### Withdrawal criteria

Participation in the study is voluntary. There are no negative consequences for non-participation or dropping out. Individuals who refuse participation or drop out get medical and psychosocial care as usual. Although an increase of suicidality is not expected during treatment, participants will be withdrawn from the study by the research team in case of heightened suicidality.

#### Informed consent

The objects and goals, detailed information about assessment and therapy as well as the procedure of randomisation will be explained to the subjects. The information will also be given in printed form for the subjects. Written informed consent will be obtained from all participants prior to inclusion. Participants who cannot give their informed consent will not be included in the study.

#### Sample size

Previous studies on PTSD treatments found a large mean effect size for PTSD outcomes (controlled *d* = 0.8 with a supportive control group) [26]. Studies on LRT also found a large mean (pre-post) effect size in older individuals with a mental disorder (in most studies depression) (g = 1.3; 19). We rest our sample size calculation on the conservative assumption of a medium-to-large effect size (d = 0.7, corresponds to f = 0.35). The sample size was calculated using G*Power 3.1.0 [27]. Given an α = 0.05 and a test power 1-β = 0.80, a total sample size of *n* = 50 (each group *n* = 25) is required. We assume a rather conservative drop-out rate of about 40% from enrolment to follow-up. Thus, we propose to start with 80 participants at enrolment (i.e. 40 in the LRT and 40 in the control group).

### Randomisation

After providing informed consent and receiving a baseline assessment, the participants will be individually assigned to a group at random. The allocation ratio for randomisation into either the treatment or control group is 1:1. The randomisation will be performed by a randomization list that was generated by a computer programme (RandList) beforehand, independently operated by the principal investigator at the University of Siegen, Germany, who does not have any personal contact with the participants. The results of the randomization will be sent to the study coordinator in Jerusalem, Israel, who will contact the therapist to explain the next steps.

### Interventions

#### Life-review therapy

The LRT-HS applied in this study includes six modules.

1. Module 1, “Introduction and motivation, diagnostics and goal setting”, includes an introduction into life-review therapy (i.e. rationale, tools, life book), an assessment of symptoms, diagnostics and individualized goal setting.
2. Module 2, “Structured life review”, is one of two the main parts of the therapy. The structure and content of this life review is adapted from that presented by Haight and Haight [22], which has already been applied with older trauma patients [21]. Four phases can be distinguished: childhood (2 sessions), adolescence (1 session), adulthood (2 sessions) and integration (2 sessions).
3. Module 3, “Narrative confrontation with the stressor”, is the second of the two main parts of the therapy. This module is positioned in between the sessions of module 2, exactly *before* the session in which the life phase is discussed in which the traumatic event has happened. If the Holocaust happened during the childhood, module 2 starts with the early childhood (birth until Holocaust), followed by module 3 (Holocaust experiences), and followed by the rest of module 2. This form of narrative exposure has been proven to be effective in the treatment of PTSD [13, 21, 28].
4. Module 4, “Examining stuck points”, continues with cognitive aspects of the integration sessions of the life review. It addresses dysfunctional thoughts that the patient often ruminates about and helps him/her to change them into helpful ones. Two strategies are used: Socratic dialogue in order to help the patient to challenge dysfunctional thoughts and formulate new, helpful thoughts, and a letter to the child/adolescent/young adult that the patient has been when he/she experienced the Holocaust.
5. Module 5, “Recapturing life”, focuses on changes in behaviour (social contacts, pleasant activities) that have already been stimulated by life review and are continued in this module.
6. Module 6, “Closing the therapy”, is the final session with a review of the therapy sessions and a planning of the next steps after therapy.

Session duration is usually 50 min (most sessions) or 60–90 min (module 3). The interventions are described in Table 1 in more detail.

**Table 1:** Description of sessions

Session no.	Topic	Therapeutic strategies
Module 1: Introduction and motivation, diagnostics and goal setting		
1	Introduction and motivation	Introduction to and rationale of life review; tools (photos and other objects); life book; organizational issues
2	Diagnostics	Diagnostic investigation of symptoms, resources and social factors; overview over biography (“timeline”)
3	Goal setting	Setting individual goals; preparation for first life review session
Module 2: Structured life review		
4–5	Childhood	Questions on childhood; life book
6	Adolescence	Questions on adolescence; life book
7–8	Adulthood	Questions on adulthood; life book
9–10	Integration	Summary and integration questions
Module 3: Narrative confrontation with the stressor		
11	Pre-Holocaust experiences	Getting an overview about the experiences; clarifying the context of a single event; details of a single event; ending the narrative; making meaning of the event (often in the following session)
12–13	Holocaust experiences	Getting an overview about the experiences; clarifying the context of a single event; details of a single event; ending the narrative; making meaning of the event (often in the following session)
14	Post-Holocaust experiences	Getting an overview about the experiences; clarifying the context of a single event; details of a single event; ending the narrative; making meaning of the event (often in the following session)
Module 4: Examining stuck points		
15	Identifying stuck points	Identifying functional and dysfunctional thinking in four domains; maybe beginning the discussion of dysfunctional thoughts
16	Discussing dysfunctional thoughts	Challenging dysfunctional thoughts (e.g. Socratic dialogue); formulating functional thoughts; introducing the exercise of writing a letter to the child-self
17	Letter to the child-self	Reading the letter out loud; summarising the most important insights and conclusions for the future
Module 5: Recapturing life		
18	Social contacts	Sharing biographic events and insights; reading the trauma narrative to relatives; maybe handing over the biography to relatives
19	Pleasant activities	Individual list of pleasant activities; planning activities; discussing problems with pleasant activities
Module 6: Closing the therapy		
20	Concluding session	Re-examining goals; evaluation of interventions; planning the future

Standardization of procedures: Therapists use a manual of this LRT-HS, which includes descriptions of the content of each session and material for the sessions. Because of the research setting, therapists will document what happens during the sessions in detail on checklists, i.e. which interventions they had applied and whether (and why) they had stressed one point more than another. Working sheets and interview guidelines are included in the manual as well. Adherence to the treatment manual is ensured by the intensive training before the intervention phase, the regular supervision and the examination of the session documentations.

Psychotherapists: The LRT-HS will be administered by trained psychotherapists, i.e. psychologists, psychiatrists and clinical social workers. All psychotherapists have a master’s degree in psychology, medicine or clinical social work. Furthermore, all participating psychotherapists will be trained for this intervention by a panel of experts and supervisors in the field of psychotherapy for older age (Dr. E. van der Hal, Dr. M. Auerbach, Dr. D. Brom, Dr. S. Forstmeier). A two-day workshop will precede the intervention phase. Supervision sessions will take part every month (i.e. about 5 sessions per patient). All sessions will be documented in a structured form and will be reflected upon in the supervision sessions.

Pilot data: We applied the described treatment programme with a few participants and found that they accepted it and rate the treatment as very helpful. In addition, symptoms were reduced to a clinically significant degree.

#### Supportive control group

Supportive control groups are often used in PTSD treatment studies because they offer both a treatment (in contrast to a waiting list control group) and at the same time are less effective than state-of-the-art treatments (between d = 0.8; 26). We implement a control group with social support in a group format that has a long tradition in all Amcha centres. As described above, it is called “social club” and has the goal of exploring and strengthening the participants’ individual and social resources when talking about current interpersonal problems, personal decisions and plans and hopes for the future. It is a meeting place which answers the need to meet other people and take part in social interaction. It provides activities like films, lectures, courses in art, music, memory improvement, gymnastics, languages (English, Yiddish), visits to interesting places, birthday celebrations, discussions of relevant issues, etc. Importantly, there are no trauma-focused interventions in the supportive group. This is a fundamental difference to the treatment group. Taken together, participants in the control group receive supportive attention, which is ethically acceptable since the participant’s need to talk and relate is met. At the same time, using this control group is methodologically appropriate because the likelihood of a treatment effect is expected to be smaller than that in the LRT group. Moreover, recruitment of study subjects will not be compromised when using this control group. Members of the staff of Amcha, who are independent of the LRT interventionists, will provide the control intervention. Control group members may receive LRT or other psychotherapy after the follow-up assessment.

Records are kept in regard to which social club meetings each participant (treatment and control group) has attended in order to be able to monitor the type and frequency of social club meetings.

### Assessment

#### Eligibility screening

Before inclusion into the study, diagnostic interviews are conducted by student research assistants with experience and training in the assessment of PTSD, depression, anxiety disorder, prolonged grief disorder and related disorders, and in the administration of structured interviews. The following self-report measures are used in the context of a clinical interview:

- PTSD: The PTSD Checklist for the “Diagnostic and Statistical Manual of Mental Disorders 5” (DSM-5) (PCL-5) is used [29]. It is a 20-item self-report measure that assesses the 20 DSM-5 symptoms of PTSD. It also makes it possible to come to a probable PTSD diagnosis according to DSM-5 criteria [30]. The questionnaire has been found reliable and valid in an Israeli sample [31].
- Depressive disorder: The Patient Health Questionnaire (PHQ-9) is a widely used 10-item self-report measure of depressive symptoms with well-established psychometric properties [32]. It makes it possible to diagnose a probable major and minor depressive disorder [33] and is sensitive to change [34]. The Hebrew version has also been found to be reliable and valid [35].
- Prolonged grief disorder is diagnosed according to the criteria proposed for the “International Classification of Diseases 11” (ICD-11) [36]. The validated Hebrew version is used [37].

To screen for potential cognitive impairment, the MMSE [38] in its Hebrew version [39] is used. The clinical work prior to registration will include anamnesis, family history, psychiatric, neurological, internal status and neuropsychological testing (MMSE).

#### Study outcome measures (primary and secondary endpoints)

All scales are applied in Hebrew language and have been tested for validity and reliability in this language. Primary endpoints are PTSD and depressive symptoms:

- PTSD symptoms: The PTSD Checklist for DSM-5 (PCL-5) is used [29] in its Hebrew version [31], a 20-item self-report measure that assesses the 20 DSM-5 symptoms of PTSD.
- Depressive symptoms: The Patient Health Questionnaire (PHQ-9) and the Geriatric Depression Scale (GDS) are used. The PHQ-9 is a widely used 10-item self-report measure of depressive symptoms with well-established psychometric properties [32] in its Hebrew version [35]. The GDS is a 15-item self-report measure developed for use with older people [40]. It has been found reliable and valid in an Israeli sample [41].

Secondary endpoints are:

- Grief symptoms: The ICD-11 Symptom-Diagnostic Test for Prolonged Grief Disorder (PG-ICD-11) questionnaire is a self-report measure of grief symptoms based on the proposal for the diagnostic assessment of PGD for ICD-11 [42]. It includes two core symptoms (yearning, preoccupation) and five additional symptoms (e.g. anger, survivor’s guilt). There has been research on the optimum symptom threshold [43]. The Hebrew version is used [37].
- Complex PTSD symptoms: The complex PTSD part of the ICD-11 Trauma Questionnaire [44] is used to measure complex PTSD symptoms. This 16-item measure captures four symptom clusters with two relating to affect regulation characterised by hyper-activation or deactivation, negative self-concept and disturbed relationships. The validated Hebrew version is used [37].
- Anxiety symptoms: The Depression Anxiety Stress Scale-21 (DASS-21) is a 21-item self-report questionnaire evaluating current anxiety, stress and depression levels [45] in its Hebrew version. The questionnaire has also been used in older people [46].
- Dysfunctional thoughts: The Posttraumatic Cognitions Inventory (PTCI) [47] assesses typical dysfunctional trauma-related thoughts and beliefs in the domains of negative cognitions about the self (21 items, e.g. “My life has been destroyed by the trauma”), negative cognitions about the world (7 items, e.g. “The world is a dangerous place”) and self-blame (5 items, e.g. “Someone else would not have gotten into this situation”). The questionnaire has been found reliable and valid in an Israeli sample [48].
- Reminiscence style: The Reminiscence Functions Scale (RFS) [49] is used to assess seven different reminiscence styles (boredom reduction, death preparation, identity/problem-solving, conversation, intimacy maintenance, bitterness revival, and teach/inform). A 28-item version has been shown to have the same factor structure [50]. We apply a Hebrew translation of the 28-item version [51].
- Positive well-being: The Life Satisfaction Index (LSI) [52] is used to measure positive aspects of well–being among older people. The LSI was intended to measure five components that had been identified in a review of previous measurement instruments. These include zest for life (as opposed to apathy), resolution and fortitude, congruence between desired and achieved goals, positive self-concept and mood tone. Positive well-being is indicated by the individual taking pleasure in his/her daily activities, finding life meaningful, reporting a feeling of success in achieving major goals, a positive self-image and optimism. The validated Hebrew version is used [53].
- Posttraumatic growth: The Posttraumatic Growth Inventory (PTGI) [54] measures positive change after traumatic experience on the following subscales (21 items): New possibilities, relating to others, personal strength, appreciation of life and spiritual change. The questionnaire is reliable and valid in an Israeli sample [55].

Outcome variables were assessed using these self-report instruments at three points in time (pretest, posttest, follow-up).

#### Predictors

Factors that might influence the endpoints will also be assessed:

- Age at time of trauma (young age is associated with more symptoms)
- Gender (female gender is associated with more symptoms)
- Educational level, i.e. years of school education
- Cognitive status (assuming low status is associated with more symptoms)
- Trauma severity, i.e. number of traumatic events, months/years experiencing the Holocaust, incl. Critical life events in the previous 5 years.

In addition, several sociodemographic variables will also be included in the assessment, such as age, sex, family status, education and occupation.

#### Assessment of safety

Participants will be regularly screened for suicidality. In case of increased suicidality - although not expected - participants will be taken out of the study by the research team. For safety and for keeping an overview over the study, a case report form (CRF) will be completed after each information, assessment or therapy session. The CRF contains checklists, test results, information about the participant (other therapies, medications, problems etc.), adverse events, date of the assessments and name of the investigators.

### Data analysis

#### Statistical analysis

Several analytic strategies are planned to test the efficacy of the treatment. First, analyses of variance (ANOVAs) will be conducted to compare the mean outcome in PTSD and depressive symptoms (primary endpoints) as well as the secondary endpoints. The age at the time of trauma, gender, educational level, cognitive status and trauma severity will be considered as covariates.

Secondly, individual growth curves will be analysed, i.e. rates and shapes of change will be examined, and individual growth curves will be compared to investigate differences.

Thirdly, multivariate analyses will be used to test the effect of the treatment with regard to primary and secondary outcomes. In this way, correlations between the outcome measures can be considered. Post hoc analyses will be performed following a significant overall effect.

#### Methods against bias

The proposed study requires several methods to avoid bias.

Selection bias: The randomisation procedure is the gold standard to avoid this bias. Random numbers will therefore be generated in advance. The randomisation procedure will be controlled by comparing the two groups with regard to possible confounders, i.e. age at the time of trauma, gender, educational level, diagnosis, cognitive status and trauma severity.

Detection bias: The baseline assessment is done before randomisation to avoid this bias. In addition, the therapists will not collect any outcome data at posttest and follow-up. This will be done by blinded members of the research team (clinical psychologists or psychiatrists or advanced master students in clinical psychology).

Performance bias: To counteract this bias, the standardisation procedure (manual, extensive training, documentation of sessions, supervision) is established.

Attrition bias: Statistical analysis will include techniques to minimise possible attrition bias, i.e. intention-to-treat analysis and imputing missing data. In case of drop-outs, it is intended to continue data collection whenever possible.

#### Handling of missing values and drop-outs

A complete data set is available if all three test times (pretest, posttest, follow-up) have been performed. A drop-out can occur, (1) if the participant does not complete the pretest, (2) decides after information or after randomisation against participation, or (3) starts with the treatment, but discontinues it.

If less than 20% of the items of one self-report instrument are missing, missing values are imputed by the mean of the scale of this participant. If a whole scale is missing, the value will be imputed by the expectation-maximation (EM) algorithm.

#### Data handling

There are three types of data that is developed or collected in this study: the LRT manual, the “life book” and the assessment data (pre, post and follow-up). The LRT manual will be published by an international publisher in English with the aim that it will be used throughout the world in the treatment of Holocaust survivors and other older, multiply traumatised individuals.

The life book is a folder that the patient fills with photos, important written (positive and negative) memories from all life phases, the trauma narratives, helpful thoughts, personal and social strengths, information about the family etc. This life book will remain in the possession of the patients and will *not* be analysed. It is a by-product of the therapy. However, if the patient wishes, he/she can submit parts of this life book as a testimony to Yad Vashem (www.yadvashem.org), an Israeli institution that preserves a database of Holocaust victims and their testimonies. The participants might wish to pass information on his/her biography to the public, but we do not request it, it is the autonomous decision of each individual Holocaust survivor.

The assessment data (information on symptoms) must also be considered very sensitive data. Therefore, the data will be stored in an anonymous form. Storage and backup will be ensured during the project by the principal investigator in cooperation with the representative of the IT department of the Centre for Information and Media Technology (ZIMT) of the University of Siegen. The long term archiving will be done in the data repository of the ZIMT.

The data and all associated documentation will be stored for a minimum of 10 years after the completion of the study, including the follow-up period.

### Communication between German and Israeli teams

We plan to communicate between the German and Israeli team in three ways. First, we plan monthly skype meetings to discuss ongoing, day-to-day issues such as recruitment and data entry. Second, there will be one face-to-face meetings per year, either in Germany or in Israel. These meetings will be used to discuss questions that have emerged in relation to supervision, adherence to the treatment manual, training of new student assistants in data entry, interpretation of pre-post analyses and pre-follow-up analyses etc. Third, data exchange between Israel and Germany is ensured via the web-based data entry system.

### Regulatory issues

This is an RCT with individuals in need of special protection and, thus, requires particular ethical considerations.

#### Ethics approval

The approvals of the ethics committee of the German Association of Psychology (Deutsche Gesellschaft für Psychologie, DGPs, 11/01/2016, ref.: SF_112015) and the ethics committee of the Hebrew University of Jerusalem, School of Social Work (15/05/2017) have been obtained. The study will be conducted in accordance with principles enunciated in the current Declaration of Helsinki, the guidelines of good clinical practice (GCP) issued by the International Conference on Harmonisation (ICH) and the German and Israeli regulatory authority’s requirements.

Participation in the study is voluntary. There are no negative consequences for non-participation or dropping out. Individuals who refuse participation or drop out will get medical and psychosocial care as usual. While ethical problems might arise due to randomisation, the control group is a social support group, so participants in both study groups will benefit if they enrol in the study.

#### Consent

Consent to joining the study will be requested from each participant only after a full verbal and written explanation has been given, an information leaflet offered and time allowed for consideration; such declarations of consent will be signed. The right of the participant to refuse to participate without giving reasons will be respected.

After the participant has entered the study, the clinician will remain free to provide alternative treatment to that specified in the protocol at any stage if he/she feels it is in the participant’s best interest, but the reasons for doing so will be recorded. In these cases, the participants remain within the study for the purposes of follow-up and data analysis. All participants are free to withdraw at any time from the treatment without giving reasons and without prejudicing further treatment.

#### Confidentiality

The principal investigator will preserve the privacy of participants taking part in the study and is registered under the Data Protection Act. Assessment and processing of all personal data is done pseudonymised, i.e. with a number and without indication of the participant’s name. A coding list on paper, which is accessible only to the study management, is used to link names with numbers. It will be destroyed after termination of data collection, whereupon the data will become anonymised.

#### Audits

The study may be subject to inspection and audit by regulatory bodies to ensure adherence to GCP, national law, and regulatory requirements.

#### Adverse events

Adverse events (AEs) are any negative medical or psychological occurrence in the study subject, e.g. worsening of symptoms. Serious adverse events (SAEs) are any negative and unexpected medical or psychological occurrence or effect that: results in death; is life-threatening (refers to an event in which the subject was at risk of death at the time of the event; it does not refer to an event which hypothetically might have caused death if it were more severe); necessitates hospitalisation, or prolongation of existing inpatients’ hospitalisation; results in persistent or significant disability or incapacity; is a congenital anomaly or birth defect.

Medical or psychological judgement will be exercised in deciding whether an AE is serious in other situations. Important AEs that are not immediately life-threatening or do not result in death or hospitalisation, but may jeopardise the subject or may require intervention to prevent one of the other outcomes listed in the definition above, will also be considered serious.

Reporting procedures: All adverse events will be reported. Depending on the nature of the event, the reporting procedures below will be followed. Any questions concerning adverse event reporting must be directed to the principal investigator in the first instance.

- Non serious AEs: All such events, whether expected or not, will be recorded.
- Serious AEs: An SAE form will be completed and faxed to the principal investigator within 24 h. However, hospitalisations for elective treatment of a pre-existing condition do not need reporting as SAEs.

The principal investigator will assess whether the event is:

- ‘Related’, i.e. resulted from the administration of any of the research procedures; and
- ‘Unexpected’, i.e. an event that is not listed in the protocol as an expected occurrence.

Reports of suspected related and unexpected SAEs (SUSARs) will be submitted within 15 days or within 7 days for fatal or life-threatening SUSARs, respectively, of the investigator becoming aware of the event to the Ethics Committee.

## Discussion

Life-review therapy for Holocaust survivors (LRT-HS) was developed to bring together LRT and a trauma-focused approach used in CBT treatment of PTSD. While LRT serves as an age-specific treatment approach for older people which meets their need to review their own life, to evaluate gains and losses and to find meaning, and was found to be very effective as a treatment for depression in old age [19] as well as PTSD [21], the trauma-focused approach aims at reconstructing the memories of traumatic events and transforming them into a coherent narrative [56]. The fragmentation of trauma-related memory hinders traumatised older people to review their lives. LRT-HS offers a framework to process trauma-related memories and integrate them into a coherent life story.

This RCT is, to our knowledge, the first to investigate a psychotherapy programme for Holocaust survivors, and the third to investigate a PTSD treatment approach in old age. The two previous RCTs investigated an internet-based writing therapy called Integrative Testimonial Therapy (ITT) with WWII survivors combining cognitive-behavioural and life review elements [8, 9] and a prolonged exposure therapy with older veterans with military service-related PTSD [10, 11]. While the latter did not try to modify the prolonged exposure approach to this age group, ITT integrates trauma-focused and life review elements, which is similar in our approach. The most important differences between LRT-HS and ITT are the setting (face-to-face vs. internet-based writing), the intensity, i.e. number of sessions (20–25 vs. 11), and the target group (multiply traumatised Holocaust survivors vs. German WWII survivors).

This RCT will not only inform us of the efficacy of this treatment approach with Holocaust survivors, it also has the potential to be applied to other multiply traumatised older people. Civil war refugees will eventually get old and need an age-adequate trauma treatment. There is already the idea to combine LRT and exposure therapy to veterans [57]. But also aging survivors of traumatisation in childhood or adolescence will become a larger group requiring psychotherapy as society is aging. Thus, the results of this study will have implications for the treatment and rehabilitation of patients many years after traumatic events.

## Supplementary information

**Additional file 1.** SPIRIT 2013 Checklist: Recommended items to address in a clinical trial protocol and related documents This table includes the page number on which the items of the SPIRIT checklist were addressed

## Data Availability

The research group will publish data generated from this study in peer reviewed journals. The dataset which will be used and/or analysed during the current study will be available from the corresponding author on reasonable request after the publication of the results. Trial registry will be updated if protocol modifications are made.

## Abbreviations

AE: Adverse event
ANOVA: Analyses of variance
CBT: Cognitive behaviour therapy
CRF: Case report form
DASS-21: Depression anxiety stress scale-21
DGPs: Deutsche gesellschaft für psychologie
DSM-5: Diagnostic and statistical manual of mental disorders 5
EM: Expectation-maximation
GCP: Good clinical practice
GDS: Geriatric depression scale
ICD-11: International classification of diseases 11
ICH: International conference on harmonisation
ITT: Integrative testimonial therapy
LRT: Life review therapy
LRT-HS: Life review therapy for Holocaust survivors
LSI: Life Satisfaction Index
MMSE: Mini Mental Status Examination
PCL-5: PTSD checklist for DSM-5
PDT: Psychodynamic therapy
PG: Prolonged grief disorder
PHQ-9: Patient health questionnaire 9
PTGI: Posttraumatic growth inventory
PTCI: Posttraumatic cognitions inventory
PTSD: Posttraumatic stress disorder
RCT: Randomised controlled trial
RFS: Reminiscence functions scale
SAE: Serious adverse event
SUSAR: Suspected related and unexpected SAE
WWII: Second world war
ZIMT: Centre for information and media technology
